# Methyl Jasmonate Affects Photosynthesis Efficiency, Expression of *HvTIP* Genes and Nitrogen Homeostasis in Barley

**DOI:** 10.3390/ijms21124335

**Published:** 2020-06-18

**Authors:** Marzena Małgorzata Kurowska, Agata Daszkowska-Golec, Monika Gajecka, Paulina Kościelniak, Wojciech Bierza, Iwona Szarejko

**Affiliations:** Institute of Biology, Biotechnology and Environmental Protection, Faculty of Natural Sciences, University of Silesia in Katowice, Jagiellońska 28, 40-032 Katowice, Poland; agata.daszkowska@us.edu.pl (A.D.-G.); monika.gajecka@us.edu.pl (M.G.); pkoscielniak@man.poznan.pl (P.K.); wojciech.bierza@us.edu.pl (W.B.); iwona.szarejko@us.edu.pl (I.S.)

**Keywords:** jasmonate, methyl jasmonate, aquaporins, tonoplast intrinsic proteins, photosynthesis efficiency, Oxygen-Evolving Complex, HvMYC2, nitrogen content, gene expression analysis, barley

## Abstract

Jasmonates modulate many growth and developmental processes and act as stress hormones that play an important role in plant tolerance to biotic and abiotic stresses. Therefore, there is a need to identify the genes that are regulated through the jasmonate signalling pathway. Aquaporins, and among them the Tonoplast Intrinsic Proteins (TIPs), form the channels in cell membranes that are responsible for the precise regulation of the movement of water and other substrates between cell compartments. We identified the *cis*-regulatory motifs for the methyl jasmonate (MeJA)-induced genes in the promoter regions of all the *HvTIP* genes, which are active in barley seedlings, and thus we hypothesised that the *HvTIP* expression could be a response to jasmonate signalling. In the presented study, we determined the effect of methyl jasmonate on the growth parameters and photosynthesis efficiency of barley seedlings that had been exposed to different doses of MeJA (15–1000 µM × 120 h) in a hydroponic solution. All of the applied MeJA concentrations caused a significant reduction of barley seedling growth, which was most evident in the length of the first leaf sheath and dry leaf weight. The observed decrease of the PSII parameters after the exposure to high doses of MeJA (500 µM or higher) was associated with the downregulation of *HvPsbR* gene encoding one of the extrinsic proteins of the Oxygen Evolving Complex. The reduced expression of *HvPsbR* might lead to the impairment of the OEC action, manifested by the occurrence of the K-band in an analysis of fluorescence kinetics after MeJA treatment as well as reduced photosynthesis efficiency. Furthermore, methyl jasmonate treatment caused a decrease in the nitrogen content in barley leaves, which was associated with an increased expression the four tonoplast aquaporin genes (*HvTIP1;2*, *HvTIP2;2*, *HvTIP4;1* and *HvTIP4;2*) predicted to transport the nitrogen compounds from the vacuole to the cytosol. The upregulation of the nitrogen-transporting *HvTIPs* might suggest their involvement in the vacuolar unloading of ammonia and urea, which both could be remobilised when the nitrogen content in the leaves decreases. Our research provides tips on physiological role of the individual TIP subfamily members of aquaporins under methyl jasmonate action.

## 1. Introduction

Jasmonates modulate many growth and developmental processes of plants including root, shoot and leaf growth, trichome and tuber formation, fruit ripening, leaf senescence pollen maturation and formation of secondary metabolites such as alkaloids and taxol [1,2,3]. Moreover, jasmonates act as stress hormones that play an important role in plant response to biotic and abiotic stresses [4,5,6]. It was postulated that the adaptive responses to environmental challenges are connected with the jasmonate-mediated changes in gene expressions [7]. In most plants, jasmonic acid (JA) and its derivates are synthesised from α-linolenic acid, although some plants may use a 16-carbon fatty acid as an initial substrate [8]. JA biosynthesis pathway is initiated in chloroplasts, then is continued in the peroxisome and finally in the cytosol [9]. Jasmonic acid and its derivates, such as jasmonyl isoleucine (JA-Ile), methyl jasmonate (MeJA) and other oxylipins, are collectively referred to as jasmonates [5] and among them MeJA was first isolated from the jasmine flower (*Jasminum grandiflorum*) [10]. The bioactive form in plants is represented by conjugate of JA with isoleucine (JA-lle) that acts as a ligand for JA receptor [11,12]. The biological activity of MeJA was only apparent when MeJA was converted to JA followed by its conjugation to JA-Ile [13].

Jasmonate signal transduction is a complicated process and its most important components are: JA receptor COI1 (Coronatine Insensitive 1) [14], JAZ proteins (Jasmonate ZIM domain proteins) that act as negative regulators in JA-induced gene expression [15,16,17], co-repressor TPL (Topless), adaptor protein NINJA (Novel Interactor of JAZ) [18] and JAR1 (Jasmonoyl Isoleucine Conjugate Synthase 1) that is an essential enzyme in generating JA-Ile—the ligand of the receptor [19]. Under stress conditions, the presence of bioactive JAs leads to the degradation of JAZ proteins, which allows the release of positively acting transcription factors, such as MYC2, that bind to JA-responsive elements in promoters of JA-responsive genes, thereby initiating their transcription [18,20]. The degradation of JAZ proteins leads to the release of JAs pathway inhibition. On the contrary, in the absence of bioactive JAs, the JAZ proteins block the MYC2 activity by recruiting the general corepressors TPL and TPL-related proteins through interaction with the NINJA, thereby blocking transcription of JA-responsive genes [20] 

The high throughput transcriptome methods, such as microarray and RNA-seq analysis conducted in Arabidopsis (*Arabidopsis thaliana*), dahurian larch (*Larix gmelinii)*, gentian (*Gentiana macrophylla*) and bilberry (*Vaccinium myrtillus*) enabled a massive screening of numerous genes induced by jasmonate treatment [21,22,23,24,25]. Genes upregulated by MeJA application in Arabidopsis were categorised as involved in jasmonate biosynthesis, disease and wounding responses, oxidative stress responses, senescence, primary and secondary metabolism, amino acid metabolism and cell-wall modification. In contrast, the expression of genes involved in photosynthesis, such as genes encoding ribulose bisphosphate carboxylase/oxygenase, chlorophyll a/b-binding protein and light-harvesting complex II were downregulated. Furthermore, transcript levels of abscisic acid induced genes involved in cold/drought-stress response, e.g., *RD22 (dehydration-induced protein); PIP5K* (*phosphatidylinositol-4phosphate 5-kinase*) was reduced, which suggests the antagonistic interaction between the jasmonate and abscisic acid signalling pathways [21]. In contrast to this research, in the MeJA treated bilberry (*Vaccinium myrtillus*), two important genes in ABA signalling pathway were found to be upregulated: *PYL ABA-receptor* and *ABF* (*ABA-responsive element binding factor*) [25]. In addition to transcriptome studies, the power of mutants for investigating jasmonate biosynthesis and signalling pathways have been clearly shown in many studies, which were reviewed by Browse [26].

The transduction pathways induced by jasmonates signal in response to abiotic and biotic stresses include cross-talks with many plant hormones: abscisic acid, auxin, ethylene, gibberellin and salicylic acid [9,27]. The role of jasmonates in plant tolerance to cold and freezing, salt, drought, heat and flooding stresses has been studied extensively [28,29,30,31,32]. Drought stress led to a rapid and transient increase in endogenous JA level in Arabidopsis and citrus (*Citrus* L.) [33,34]. The endogenous JA content increased after salt treatment in Arabidopsis [35] and tomato (*Lycopersicum esculentum*) [36]. Furthermore, the application of exogenous JA can efficiently mitigate the damage caused by abiotic stresses in plants. In most experiments that mimic jasmonate-mediated gene activation, JA or MeJA were used for exogenous application [21]. It was observed that MeJA treatment enhanced drought tolerance in rice (*Oryza sativa*) [32], soybean (*Glycine max*) [37] and broccoli (*Brassica oleracea*) [38]. Exogenous supply of MeJA to an ornamental plant *Malus crabapple* (Rosaceae) was protective to ozone stress-induced toxicity [39] or reduced effect of cadium-induced injury in rice seedlings [40]. It has also been reported that MeJA treatment increased postharvest chilling tolerance in fruits of tomato (*Lycopersicon esculentum*) [41], avocados (*Persea americana*) [42], blood orange (*Citrus sinensis*) [43] and pomegranate (*Punica granatum*) [44]. Exogenous MeJA improved salt tolerance in almond (*Prunus dulcis*) [45] and sea-lavender (*Limonium bicolor*) [46]. In barley (*Hordeum vulgare*), jasmonic acid-mediated adaptation to salinity stress was reported and characterised [47,48].

Aquaporins, which belong to the membrane intrinsic protein (MIP) family, form channels in cell membranes and perform various functions, of which the water channel function is best described [49]. Aquaporins have been classified into five subfamilies referred to as plasma membrane intrinsic proteins (PIPs), tonoplast intrinsic proteins (TIPs), nodulin 26-like intrinsic proteins (NIPs), small and basic intrinsic proteins (SIPs) and the uncategorised X intrinsic proteins (XIP) [50]. The amino acid sequences of all aquaporins contain four positions in which amino acids form so-called aromatic/arginine (ar/R) selectivity filter, and based on its structure the substrate or substrates transported by the aquaporin are predicted [51]. The tonoplast intrinsic proteins (TIP) that are located mainly in the tonoplast facilitate the rapid osmotic equilibration between a vacuole and a cytosol [52]. TIPs are responsible for the precise regulation of the movement of water but also substrates other than water, such as ammonia, formamide, hydrogen peroxide, urea and glycerol [53]. The transports of these substrates were predicted by bioinformatic analysis and some of them were also proved experimentally. It was found that barley HvTIP1;1, HvTIP1;2 and HvTIP2;3 were capable of transporting water when they were expressed in yeast [49]. In turn, experiments with *in vitro* expression of Arabidopsis AtTIP1;1, AtTIP1;2, AtTIP2;1 and AtTIP4;1 in yeast or oocytes showed that they are capable of transporting urea and thus they might play a role in equilibrating urea concentration between different cellular compartments [54,55]. Furthermore, the expression of AtTIP2;1, AtTIP2;3 and TaTIP2;2 from wheat (*Triticum aestivum*) were found to transport ammonia when they were expressed in yeast or oocytes [56,57,58]. In barley (*Hordeum vulgare*), 11 members of TIP subfamily have been identified [53] and seven of them exhibited significant expression changes in leaves of drought-exposed plants [59].

Barley is one of the most important crop species that ranks fifth in worldwide production (tonnes) and area harvested (ha) [60]. It is grown in many regions across the world and in more than 100 countries [61]. Barley grains are used as human food, animal feed and a raw material for the malting and brewing industry. Due to the fact that barley genome sequence has been assembled [62] and that this species is well adopted to the extreme environmental conditions, barley can serve as a model crop in studying plant response to abiotic stresses [63].

In our previous studies on the role of aquaporin genes in drought stress response in barley, we identified two *cis*-regulatory elements (G-box: CGTCA and TGACG) in the promotor regions of eight *HvTIP* genes [59]. These motifs are characteristic for the methyl jasmonate-induced genes [64,65,66]; thus, we hypothesised that HvTIPs could be involved in response to the jasmonate signalling. To the best of our knowledge, no studies have been carried out on the impact of methyl jasmonate on the aquaporin expression in plants. Such a study could lead to identification of new targets regulated by this phytohormone, which might be involved in the maintenance of water and/or nitrogen homeostasis, and contribute to the growth inhibition caused by MeJA.

The interactions between plant aquaporins and photosynthesis has been described mainly for PIP (plasma membrane intrinsic protein) subfamily in different plant species [67,68]. The role of aquaporins during photosynthesis proved their function in transporting water and CO_2_ across the membrane of chloroplasts and thylakoids [69]. Some of TIP subfamily members are localised not only in tonoplast, but also in chloroplast and thylakoid membranes. Such a location was detected for TIP1;1, TIP1;2 and TIP2;1 in Arabidopsis [70,71,72]. It was also shown that overexpression of citrus (*Citrus limonia*) gene *CsTIP2;1* in transgenic tobacco (*Nicotiana tabacum*) led to improvement of photosynthetic capacity under drought stress [73].

In the present study, we examined the effect of exogenous methyl jasmonate on the expression profiles of *tonoplast intrinsic protein* (*TIP*) genes in barley. First, we determined the influence of methyl jasmonate on growth parameters and photosynthesis efficiency of barley seedlings exposed to different MeJA doses in hydroponic solution. To confirm the physiological data, we analysed the expression profiles of genes encoding the extrinsic proteins of Oxygen-Evolving Complex (OEC) and the *HvMYC2* gene that encodes a key transcription factor involved in jasmonate response. The analysis of transcription activity of eight *HvTIPs* after MeJA treatment indicated that jasmonates induce the expression of specific *HvTIPs* which might be engaged in ammonia and urea remobilisation in barley leaves. We discuss the role of MeJA-responsive aquaporins in regards to water and nitrogen homeostasis under stress conditions pointing at the need for further studies, preferably using mutants and/or overexpression lines, to elucidate the exact roles of individual TIPs.

## 2. Results

### 2.1. Methyl Jasmonate Treatment Led to Growth Reduction of Barley Seedling

**Figure 1:**
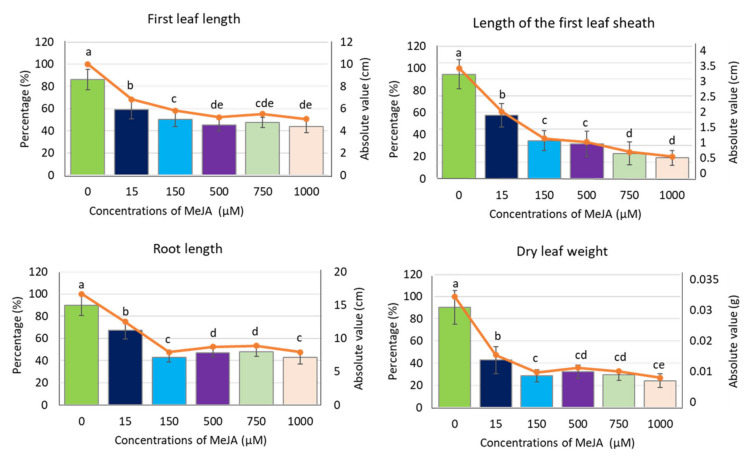
Growth parameters of barley seedling cv. ‘Sebastian’ after MeJA treatment. The parameters are presented as the absolute values (in centimetres or grams) and as a percentage of control, where 100% is the value for the seedling growth parameters without MeJA (control). Each of the values presented are the means ± SD, ten plants per one biological replication, three biological replications were used. Statistically significant differences between different MeJA concentrations were assessed using a one-way ANOVA followed by the Fisher Least Significant Difference (LSD) test (*p* < 0.05) and are indicated by different letters.

To analyse the effect of exogenous methyl jasmonate (MeJA) on barley seedling growth and physiology, we applied a five-day treatment of spring barley cv. “Sebastian” with different concentrations of MeJA in a hydroponic system (Figure S1). Control seedlings were grown simultaneously in a ½ Hoagland’s solution without supplement of the phytohormone. All applied MeJA concentrations (15, 150, 500, 750 and 1000 µM) caused a significant reduction of barley seedling growth, noticeable in all analysed parameters: length of the first leaf, length of the first leaf sheath, root length and dry leaf weight (Figure 1). Among these parameters, the length of the first leaf sheath and dry leaf weight showed the highest reduction already after treatment with 150 µM (63% and 68% reduction, respectively). This concentration of MeJA led also to approximately 50% reduction of other growth parameters, i.e., leaf and root length, while the higher MeJA concentrations did not cause further growth reduction, except for the first leaf sheath length (Figure 1). This growth parameter can be pointed out as the best indicator of MeJA influence on barley seedling growth in hydroponic solution.

### 2.2. Methyl Jasmonate Treatment Reduced the Efficiency of Photosynthesis in Barley Seedlings

**Figure 2:**
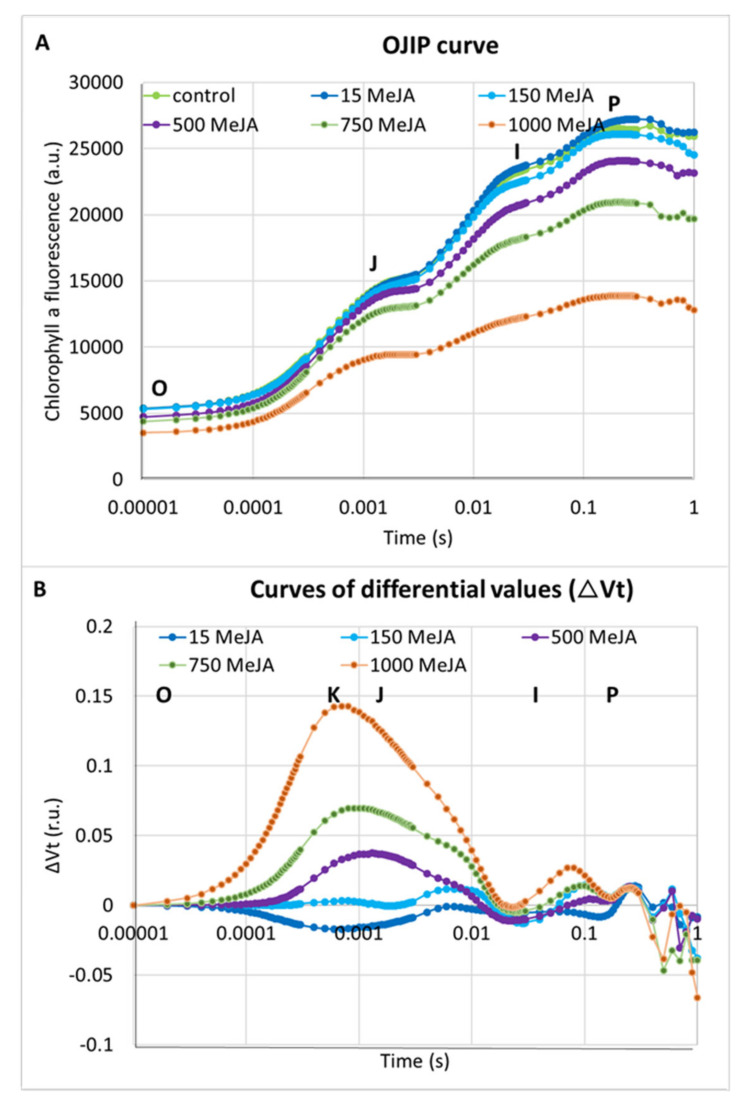
The effect of different concentrations of MeJA on the photosynthesis efficiency in seedlings of barley cv. ‘Sebastian’: (**A**) OJIP induction curves after treatment with MeJA; and (**B**) differences in the relative variable fluorescence of the O-P-normalised induction curves (∆Vt) after MeJA treatment. The values are presented as the means ± SD, ten plants per one biological replication, three biological replications were used. The ∆Vt curves were constructed by subtracting the normalised fluorescence values (between O and P) recorded in control conditions from those recorded after MeJA treatment.

In addition to seedling growth parameters, we analysed the effect of all applied concentrations of methyl jasmonate on photosynthesis efficiency. The chlorophyll *a* fluorescence (ChIF) analysis was used to compare the response of barley seedlings to MeJA treatments. We applied the JIP-test to track changes in fluorescence kinetics [74]. Four phases occur typically in the analysis: O, J, I and P. The O-J (0–3 ms) phase reflects the gradual reduction of the photosystem II (PSII) acceptor side. The J-I (3–30 ms) phase describes a reduction of the plastoquinone (PQ) pool. The I-P (30–200 ms) phase is connected to the final reduction of the electron acceptors of the photosystem I (PSI): PC (plastocyanin), P700 (photosystem I), the Fe/S (iron-sulfur) clusters and Fd (ferredoxin) [75]. Higher MeJA concentrations induced changes in photosystem designated by occurrence of additional peak—a K-band. In the case of the OJIP curve analysed after 1000 µM of MeJA treatment, the K-band became dominant (Figure 2A,B).

Differences between the applied concentrations of MeJA were most distinguished when the performance index per absorption (PI_ABS_) was analysed. PI_ABS_ provides quantitative information about the general state of plants and their vitality. PI_ABS_ is characterised by the following parameters: the absorption of light energy (ABS), trapping of excitation energy (TR) and conversion of excitation energy to electron transport (ET) [75]. Interestingly, the lowest MeJA concentration (15 µM) led to an 18% increase of the PI_ABS_ parameter when compared to control plants (Table 1). Additionally, 15 µM of MeJA caused a 10% increase in the number of active reaction centres per illuminated cross-section (RC/CS) compared to the control plants. On the contrary, the PI_ABS_ was profoundly lower after five days of seedling treatment with the highest MeJA concentrations, 750 and 1000 µM. The PI_ABS_ was 32% and 65% lower in seedlings treated with these doses compared to the seedlings grown in control conditions (Table 1). RC/CS was also 29% and 64% lower after treatment with 750 and 1000 µM MeJA compared to control plants. The values of parameters in a cross-section of the leaf area such as ABS/CS, TR_0_/CS, Et_0_/CS and DI_0_/CS decreased after treatment with 500, 750 and 1000 µM of MeJA. The highest concentration caused a vast decrease in these parameters—54% on average (Table 1). Taken together, these results indicate a strong influence of MeJA on the action of the photosynthetic apparatus in barley seedlings after treatment with the higher concentrations of phytohormone, 500–1000 µM for five days. We selected the concentration of 500 µM of MeJA that caused a strong growth reduction (Figure 1), a noticeable effect on the photosynthesis efficiency (Table 1) and a K-band occurrence during fluorescence kinetics analysis (Figure 2A,B) for further molecular analysis.

To gain more insight into the influence of MeJA on photosynthesis efficiency, we analysed changes in expression of genes encoding the extrinsic proteins of Oxygen-Evolving Complex (OEC). Changes in the expression level of OEC proteins might be the reason for the occurrence of the K-band in analysis of fluorescence kinetics in response to MeJA treatment (Figure 2A,B). We identified four potential barley orthologues of Arabidopsis genes encoding OEC proteins, namely *HvPsbO, HvPsbP, HvPsbR* and *HvPsbQ* (*Photosystem II subunit O, P, R, Q*) (Table S1), and analysed their expression profile after treatment of barley seedlings with 500 µM for different periods of time (4–120 h).

**Figure 3:**
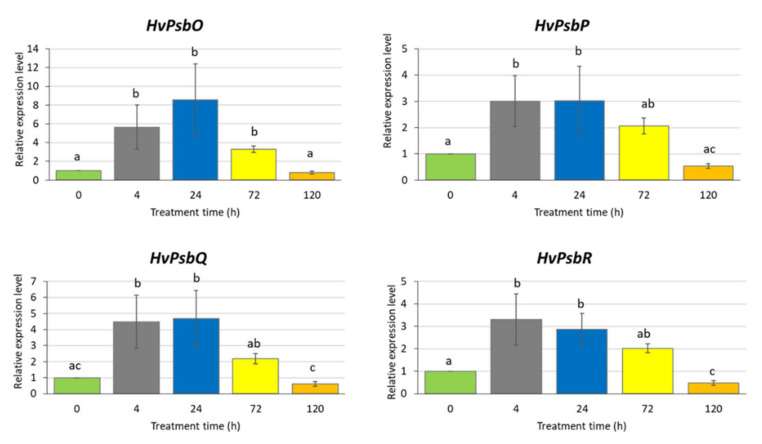
Expression of the genes encoding extrinsic proteins of Oxygen-Evolving Complex in leaves of barley seedlings treated with 500 µM methyl jasmonate. The relative expression level was assessed in relation to control plants grown without MeJA solution. The statistical analysis was performed using a one-way ANOVA followed by the Fisher Least Significant Difference (LSD). Statistically significant differences (*p* < 0.05) are indicated by different letters.

There were significant changes in the expression level of the studied genes in response to the applied MeJA treatment (Figure 3). Interestingly, we observed a similar expression profiles of all analysed genes: an increase of the expression level already after 4 h MeJA treatment (from three to six times) that remained at the same level after 24 and 72 h and finally declined after a longer exposure to MeJA (120 h). After a prolonged treatment with MeJA (120 h), the expression of three genes, namely *HvPsbO, HvPsbP* and *HvPsbQ*, returned to the level observed in control conditions while *HvPsbR* expression reduced two-fold compared to the control (Figure 3). The downregulation of *HvPsbR* gene expression might lead to the reduced activity of OEC.

### 2.3. Analysis of the HvMYC2 Gene Expression after Methyl Jasmonate Treatment

**Figure 4:**
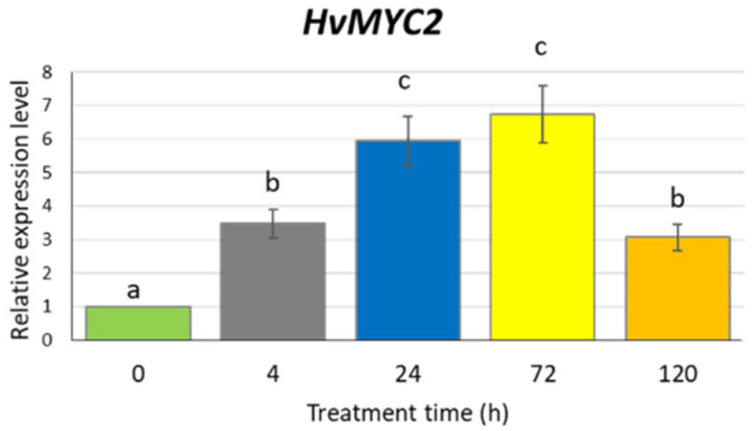
Expression of the *HvMYC2* gene in the leaves of barley seedlings after 500 µM methyl jasmonate treatment. The relative expression level was assessed to control plants grown without MeJA solution. The statistical analysis was performed using a one-way ANOVA followed by the Fisher Least Significant Difference (LSD). Statistically significant differences (*p* < 0.05) are indicated by different letters.

To confirm that our experimental conditions induce the jasmonate signalling pathway, we analysed the expression profile of the *HvMYC2* gene, a homologue of Arabidopsis *MYC2* encoding a key regulator in the jasmonate signal transduction. Barley seedlings were exposed to 500 µM MeJA for 4, 24, 72 and 120 h. At all investigated treatment points, the *HvMYC2* gene was upregulated, with a level of expression 6–7 times higher after 24 and 72 h treatment, compared to the untreated control (Figure 4). The prolonged treatment (five days) resulted in a similar transcription activity of *HvMYC2* as the 4-h treatment, i.e., higher than in control plants but significantly lower than after 24- or 72-h treatments.

### 2.4. Changes in the Expression Profiles of the HvTIP Genes after Methyl Jasmonate Treatment

Based on *in silico* analysis of promoter regions of *tonoplast intrinsic protein (TIP*) genes, we assumed that their expression might be regulated by jasmonate-inducted signalling pathway [58]. We selected eight *HvTIP* genes that are expressed in barley at the seedling stage: *HvTIP1;1, HvTIP1;2, HvTIP2;1, HvTIP2;2, HvTIP2;3, HvTIP3;1, HvTIP4;1* and *HvTIP4;2*. Among TIPs encoded by these genes, three (HvTIP1;1, HvTIP1;2 and HvTIP2;3) were experimentally proven as transporting water [48]. Moreover, based on the key structural features of the amino acid sequences of analysed HvTIPs, it was postulated that they could transport also other substrates, such as hydrogen peroxide (HvTIP1;1, HvTIP1;2, HvTIP2;1, HvTIP2;2, HvTIP2;3 and HvTIP3;1), ammonia (HvTIP2;1, HvTIP2;2 and HvTIP2;3) and urea (HvTIP1;1, HvTIP1;2 and HvTIP4;2) [52]. Only for HvTIP4;1 the transported substrate has not been predicted.

**Figure 5:**
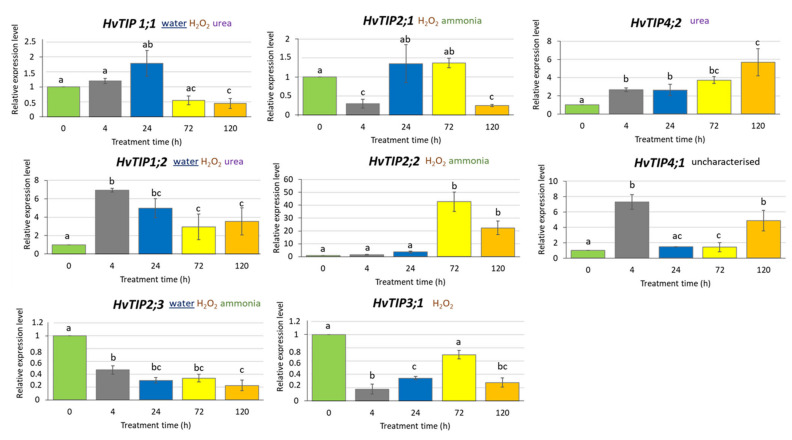
Expression profiles of *HvTIP* genes in leaves of barley seedlings treated with 500 µM MeJA in a mini-hydroponic system. The chosen potential substrate or substrates transported by HvTIPs were indicated [52]. The underlined substrate has been experimentally proven for barley HvTIPs [49]. The relative gene expression level in MeJA treated plants was normalised to the control plants grown in a ½ Hoagland’s solution. The statistical analysis was performed using a one-way ANOVA followed by the Fisher Least Significant Difference (LSD) test. Statistically significant differences (*p* < 0.05) are indicated by different letters.

To determine whether exposure to MeJA affects *HvTIP* genes expression, we analysed their transcription profile after 500 µM MeJA treatment for 4, 24, 72 and 120 h. We identified different expression patterns of *HvTIP* genes after MeJA treatment. The expression of four genes, namely *HvTIP1;2, HvTIP2;2, HvTIP4;1* and *HvTIP4;2*, increased compared to the control plants for all or at least two treatment periods (Figure 5). Among the upregulated genes, three, namely *HvTIP1;2, HvTIP2;2* and *HvTIP4;2*, are potentially involved in urea or ammonia transport, while for *HvTIP4;1* the substrate has not been determined. The transcriptional activity of only one gene, *HvTIP4;2*, increased constantly during the treatment and after 120 h achieved the highest, six times upregulated, expression level. However, the greatest increase in transcriptional activity was observed for the *HvTIP2;2* gene whose expression was more than 40-fold higher after 72-h MeJA treatment and 20-fold higher after 120-h exposure, compared to the control seedlings (Figure 5). On the contrary, among genes whose expression decreased at some time points compared to control plants were: *HvTIP1;1, HvTIP2;1, HvTIP2;3* and *HvTIP3;1*. HvTIP3;1 is predicted to transport only H_2_O_2_ while HvTIP2;1 can also transport ammonia and HvTIP1;1 and HvTIP2;3 transport water. Only for one of the downregulated genes, *HvTIP2;3*, the constant decline of expression, related to the time of MeJA exposure, was observed (Figure 5). This experiment shows that methyl jasmonate-induced signalling pathway modulates expression of *HvTIPs* in barley seedling, which is in line with the *in silico* analysis of their promotor regions. The examined *HvTIP* genes showed changes in the profile and the range of expression, suggesting that individual HvTIPs may play an important and different function in processes regulated by methyl jasmonate.

### 2.5. Nitrogen Content in Leaves of Barley Seedlings after Methyl Jasmonate Treatment

**Figure 6:**
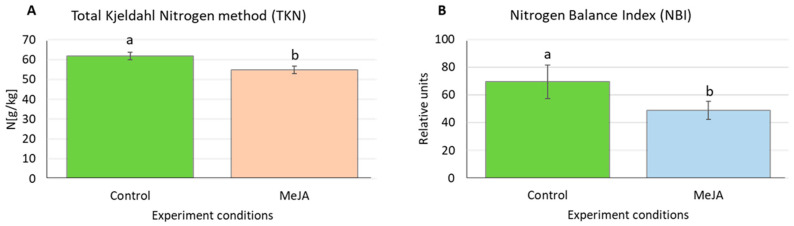
Nitrogen content in barley leaves after treatment with 500 µM MeJA for five days measured using: (**A**) Total Kjedahl Nitrogen method (TKN); and (**B**) Nitrogen Balance Index (NBI). Data are presented as the means ± SD for three biological replications, 0.5 g leaf tissue and 10 seedlings per replication in the Kjedahl and NBI method, respectively. Statistically significant differences between the control conditions and the MeJA treatment were assessed using a one-way ANOVA followed by the Fisher Least Significant Difference (LSD) test (*p* < 0.05) and are indicated by different letters.

Four aquaporin genes upregulated by MeJA treatment at all or two investigated time points, namely *HvTIP1;2, HvTIP2;2*, *HvTIP4;1* and *HvTIP4;2*, are potentially involved in transport of nitrogen compounds, urea and ammonia through tonoplast, while *HvTIP4;1* has not been characterised. To confirm that methyl jasmonate induces changes in nitrogen homeostasis, we examined the nitrogen content in barley leaves after MeJA treatment. Using two different parameters, Total Kjedahl Nitrogen (TKN) method and Nitrogen Balance Index (NBI), we demonstrated that MeJA-treated seedlings exhibited the reduced nitrogen content compared to control plants. The NBI parameter showed 36% and TKN method 11% decrease in the leaf nitrogen content after 120-h exposure to 500 µM MeJA (Figure 6). The induction of *HvTIP1;2*, *HvTIP2;2*, *HvTIP4;1* and *HvTIP4;2* by MeJA might suggest their role in ammonia and urea remobilisation from the vacuole.

## 3. Discussion

Photosystem II (PSII) is one of the most sensitive elements of the photosynthetic machinery and is highly responsive to stress conditions [76,77]. The JIP-test provides much information about the maintenance of the photosynthetic apparatus under various growth conditions. The induction of the Chl *a* fluorescence transient results in polyphasic rise, which is called an O-J-I-P fluorescence transient [74]. Our results prove that, after treatment with higher concentrations of MeJA, the photosynthesis efficiency generally decreased. We identified appearance of a K-band during analysis of fluorescence kinetics in response to MeJA concentrations from above 500 µM MeJA. This additional K-band has previously been detected in the heat-stressed leaves of potato [78], in the drought-stressed leaves of barley [75,79,80] and in the ABA-treated seedlings of barley [75]. It was suggested that the K-band is related to the destruction of the Oxygen-Evolving Complex (OEC), which further leads to the imbalance between the electron flow leaving the reaction centre (RC) towards the acceptor side and the electron flow coming to the RC from the donor side [74]. As a consequence of this impairment, the disintegration of the Mn cluster in the absence of the extrinsic proteins occurs [81]. We proved that treatment with 500 µM of MeJA for five days led to both the appearance of the K-band and the downregulation of one of the OEC genes, *HvPsbR*. This is in line with a hypothesis of Strasser et al. [74] because the decreased transcriptional activity of this gene might result in the impairment of the OEC action. It was also confirmed in the study on *Chlamydomonas reinhardtii* in which the downregulation of *PSBR* expression diminished the efficiency of oxygen evolution and the extent of nonphotochemical quenching and had an impact on the stability of the oxygen-evolving complex [82]. In turn, the studies in Arabidopsis showed that *psbR* mutants exhibited PSII conformational changes, a slower electron transfer and a lower PSII activity [83]. It was concluded that the lack of the PsbR protein modifies both acceptor- and donor-side of the PSII complex [84]. Furthermore, the functional defects observed in the *psbR* mutant were due to the defective assembly and/or binding of the PsbP and PsbQ components to the Photosystem II complex in the absence of the PsbR protein [85]. Complete elimination of PsbQ has a lesser effect on PSII function, but plants lacking PsbR or both PsbR and PsbQ are characterised by clear defects in PSII activity [86]. In barley, the downregulation of genes encoding the OEC proteins has been shown after exposure to drought stress [75]. Severe and mild water deficit caused reduction of *HvPsbO* expression in both roots and leaves of drought-stressed barley plants [87,88]. The microarray-based screening of the jasmonate-responsive genes in *Arabidopsis thaliana* showed downregulation of genes involved in the chloroplast constitution and photosynthesis [21]. Among them, *AtPsbQ1* (At4g21280) was identified.

The phytochemical activity under stress conditions is primarily regulated via the deactivation of the PSII reaction centres [89]. We demonstrated that treatment with 750 and 1000 µM of MeJA for 120 h (five days) led to the decrease in the number of active reaction centres per illuminated cross-section (RC/CS). Generally, the use of 500 µM and higher concentrations of MeJA led to the decrease of the following parameters: absorption energy flux per cross-section (ABS/CS), trapped energy flux per CS (TR_0_/CS), electron transport flux per CS (ET_0_/CS) and dissipation energy flux per CS (DI_0_/CS). This clearly indicates the reduced efficiency of photosynthesis. The same OJIP parameters were reduced in barley (*Hordeum vulgare*) by drought stress [90]. The drought mainly limited the number of active PSII reaction centers, which further led to reduce amount of trapped energy per leaf cross-section [90]. Another study in barley performed using OJIP test showed the exact pattern of changes in response to drought and rapid dehydration [75]. The drought stress caused decrease in a value of parameters derived from the fast chlorophyll *a* fluorescence kinetics and the rapid dehydration resulted in a similar pattern of OJIP curve as early drought stress [75]. The phenomenological energy fluxes for absorption (ABS/CS), trapping (TRo/CS) and electron transport (ETo/CS) were reduced in zoysiagrass (*Zoysia japonica*) when the plants were subjected to cold stress [91]. In turn, salinity stress decreased only the electron transport flux per cross section (ET_O_/CS) in sorghum (*Sorghum bicolor*) [92]. Furthermore, the macronutrient deprivation in maize (*Zea mays*) had an impact on the same OIJP parameters and decrease in ABS/CS, TR_0_/CS, ET_0_/CS and DI_0_/CS values were observed [93]. Interestingly, in our study, the lower concentration (15 µM) of MeJA applied for 120 h caused a 10% increase in the value of RC/CS compared to the control plants. We also observed upregulation of all *HvPsb* genes after a shorter exposure (4 and 24 h) to 500 µM MeJA. Although the different treatments (concentration × time) cannot be directly compared, it is worth noting that the dependence of photosynthesis activity on the MeJA dose used in the exogenous MeJA application was observed in many studies [27,94]. At a concentration of 100 µM MeJA or higher, downregulation of genes involved in photosynthesis and decrease of photosynthesis activity were detected when MeJA was applied as a spray or added to hydroponic culture [21,95]. On the contrary, at a concentration of 50 µM or lower, often combined with abiotic stress treatments, elevation of the net photosynthetic rate or photosynthetic pigments were observed [96,97]. It is well known that the level of phytohormones including jasmonic acid varies according to the tissue type, development stage and external stimuli [36,98,99,100,101]. The highest levels of JA have been reported in the fruit, flowers and young leaves, whereas much lower levels have been found in the roots and mature leaves [98,99,102]. The detail analysis of jasmonate content performed in soybean (*Glycine max*) revealed a range of 50–2000 ng/g of fresh weight, depending on the organ, and a five-fold increase in the JA levels after dehydration [99]. In our study, we added different concentrations of MeJA to a ½ Hoagland’s medium in which the barley seedlings were grown, so the phytohormone first had to be transported from the roots to leaves. Jasmonate can move readily in plants in the liquid phase or the vapor phase and can diffuse through the membranes [103,104]. To choose MeJA concentrations for molecular studies, we were looking for MeJA doses that would turn on the jasmonate signalling. We tested the expression profile of *HvMYC2* gene which encodes the key transcription factor that binds to JA-responsive elements in promoters of JA-responsive genes, and thus plays a crucial role in the JA signal transduction process. The lower MeJA concentration (i.e., 150 µM for 4- and 24-h treatment, data not shown) did not affect *HvMYC2* expression, while, after 4-h treatment with 500 µM, the *HvMYC2* transcript level increased three-fold.

It should also be noted that there are large discrepancies among the methods and range of doses which have been used for exogenous treatment with MeJA. Among applied methods of treatment there are: hydroponic systems, spraying the plants, floating leaf pieces in phytohormone solution and irrigating plants with phytohormone solution. For example, in *Hordeum vulgare*, the cut pieces of leaves were floated in 45 µM of MeJA solution for 24–72 h [105]. In *Glycine max*, 50 µM MeJA was used for leaf spraying of mature plants [106]. In *Brassica napus*, 100 µM was applied for eight-day treatment of mature plants in hydroponic solution [96], while in *Citrus*, mature plants grown in perlite were supplied with 1000 µM solution [34] and in *Brassica oleracea* seedlings were sprayed with 5000 µM [107].Under our experiment conditions the expression of *HvMYC2* gene was upregulated, indicating that jasmonate signalling was turned on. This basic helix-loop-helix transcription factor has a key position in the signal transduction process that regulates response to jasmonates [108,109]. Studies in different plant species demonstrated that MYC2 transcription was induced by MeJA treatment, e.g., in apple (*Malus x domestica*) [110] and yew-tree (*Taxus* sp.) [111].

Total Kjeldahl Nitrogen method is often used as reference method for N determination in plants [112]. Nitrogen Balance Index (NBI) used for N estimation is a not destructive method based on fluorescence measurement. It evaluates the ratio of chlorophyll to polyphenols. The chlorophyll and the polyphenols content in plant leaves are inversely dependent on the nitrogen status [113].

In our experiment, the exogenous treatment with MeJA caused reduction of nitrogen content in barley leaves. The reduction in nitrogen uptake as an immediate consequence of MeJA treatment has been described previously in *Brassica napus* and it was concomitant with a remobilisation of endogenous N from leaves to roots [95]. In our experiments, this reduction was associated with increased expression of three *HvTIP* genes that are predicted to transfer nitrogen compounds (urea or ammonia) between vacuole and cytoplasm. Among all investigated *HvTIP* genes, the highest increase in transcriptional activity was observed for the *HvTIP2;2* gene whose expression was more than 40-fold higher after 72-h MeJA treatment, compared to the control seedlings. This isoform is postulated to transport ammonia based on bioinformatic analysis in barley [53]. The function of the wheat aquaporin TaTIP2;2 was analysed in yeast. The results suggest that NH_3_ is not transported in file with water, but through a separate pore in a tetramer conformation [58]. The expression level of another gene, *HvTIP4;2*, which is postulated to transport urea, increased constantly during exposure to MeJA and reached six-fold upregulation after 120-h treatment. Furthermore, the increase of expression level after MeJA treatment at different treatment points was detected for two other *HvTIP* genes: *HvTIP1;2* and *HvTIP4;1.* Urea is one of the possible substrates transported by *HvTIP1;2* isoform, while the substrate for *HvTIP4;1* has not been predicted in barley. However, Arabidopsis homologue of *HvTIP4;1* was shown capable of transporting urea [54]. The increase in expression level of *HvTIP1;2, HvTIP2;2, HvTIP4;1* and *HvTIP4;2* after MeJA treatment might lead to vacuolar unloading of ammonia and urea from vacuole to cytoplasm in response to decreased nitrogen content in leaves observed in our study. The repression of nitrate uptake by roots and, consequently, the reduced level of nitrogen in leaves was observed in rice (*Oryza sativa*) and tomato (*Solanum lycopersicum*) after MeJA treatment [114,115].

Water is the most important substrate whose transport is facilitated by aquaporins. There are three TIP proteins in barley, which are proved to be involved in water transport by in vitro experiments: HvTIP1;1, HvTIP1;2 and HvTIP2;3 [49]. Treatment with a high dose of MeJA (500 µM × 120 h) led to downregulation of the expression of two of them—*HvTIP2;3* and *HvTIP1;1*—and the same effect was observed under drought stress [59]. The reduced expression of water-transporting aquaporin genes under drought stress could lead to the decrease in water permeability of membranes in order to avoid water loss. MeJA treatment led to the same effect as drought treatment even for plants which were grown in hydroponic culture. It should be noticed that the prediction of cell location for these two aquaporins (HvTIP2;3 and HvTIP1;1) is dual—they may be located in the tonoplast as well as in plasma membrane [53].

Contrary to *HvTIP2;3* and *HvTIP1;1* genes, the expression profile of the third water-transferring aquaporin gene (*HvTIP1;2*) differed after MeJA and drought treatment. Under drought stress, a strong downregulation of the *HvTIP1;2* was detected [59], while its expression increased after MeJA treatment. It should be noted that all three water-transporting aquaporins are also predicted to transport nitrogen compounds (urea or ammonia) and hydrogen peroxide. The similar profile of *HvTIP2;3* and *HvTIP1;1* genes after drought and MeJA treatments and different profile of *HvTIP1;2* indicate that not all aquaporin genes involved in drought response are related to the MeJA pathway.

*HvTIP3;1* encodes an isoform that is predicted to transport hydrogen peroxide only [53]. H_2_O_2_ is a relatively long-lived molecule compared with other reactive oxygen species (ROS) [116]. It is potentially harmful at high concentrations because of its high reactivity. It is known that MeJA treatment and drought lead to the generation of ROS [117,118,119,120]. Each subcellular compartment in plants including vacuole contains its own set of ROS-producing and ROS-scavenging proteins and TIP could be a part of this mechanism [119]. Under drought stress, expression of *HvTIP3;1* gene rose 5000 times compared to the optimal water conditions [59], while after MeJA treatments its activity decreased. Other *HvTIP* genes that are postulated to transport H_2_O_2_ also showed different expression profiles after drought and MeJA treatment suggesting that different aquaporins are involved in response to MeJA and drought induced oxidative stresses. Two pairs of genes, *HvTIP4;2* and *HvTIP2;3* as well as *HvTIP2;1* and *HvTIP4;1*, had opposite patterns of expression under MeJA treatment. Interestingly, the opposite patterns of expression were also detected under drought stress for *HvTIP2;1* and *HvTIP4;1* [59].

## 4. Materials and Methods 

### 4.1. Plant Material

This research was performed using the two-row spring barley cultivar “Sebastian”. This variety was created in Sejet Plant Breeding company in Denmark as a cross “Visksa” × “Lux”. “Sebastian” variety is characterised by its high yield potential, high biomass production, good brewery traits, resistance to lodging, susceptibility to mildew (*Blumeria graminis* f.sp. *hordei*) and a moderate susceptibility to scald (*Rynchosporium secalis*) and leaf rust (*Puccinia hordei*). It also has good resistance to net blotch (*Pyrenophora teres*) [121].

### 4.2. Treatment with Methyl Jasmonate (MeJA) Using a Mini-Hydroponic System

The mini-hydroponic system was used to studying the effect of methyl jasmonate (MeJA, Sigma Aldrich Cat. No. 392707) on seedling growth. The mini-hydroponic setup consisted of a 1.2 L opaque plastic container, which was covered with a lid containing 24 holes and air distributors with 12 outlets with a non-return valve attached to an air pump (Figure S1). A half-strength Hoagland’s nutrient solution was used as the medium for the treatment with MeJA and pH was adjusted to 5.8. The composition of this solution was described elsewhere [122]. The seeds were surface sterilised by soaking in the 1% sodium hypochlorite for 15 min followed by rinsing them in sterile water three times for 5 min. Then seeds were placed on three filter papers that had been soaked with 5 mL of sterile water in Petri dishes, sealed with parafilms and kept for imbibition at 4 °C for 24 h in the dark, and then transferred to 21 °C in the dark for next 48 h and another 48 h in the light at the same temperature. Five-day-old seedlings with roots and leaves were transferred into the holes of a plastic container lid covered with filter paper, 24 seedlings per container. The roots of the seedlings were immersed in the medium. One container was assumed to be one biological repetition with three repetitions per treatment. Five different concentrations of MeJA were used to investigate seedling growth (15, 150, 500, 750 and 1000 µM), and a medium without the phytohormone supplement was used in the control (Figure S1). After five days of growth, the following parameters were analysed: leaf, root, distance between seed and unrolled part of the first leaf and the dry mass of a leaf after drying at 60 °C for 24 h. Ten seedlings per one biological repetition were analysed. The experiments were conducted in a growth chamber with controlled conditions: light intensity 250 μM m^−2^ s^−1^, temperature 20 °C/18 °C (day/night) and photoperiod 16/8 h.

### 4.3. Physiological Analyses

#### 4.3.1. Chlorophyll a Fluorescence (ChIF) and Nitrogen Balanced Index (NBI) Analyses

The chlorophyll *a* fluorescence parameters were measured using a Plant Efficiency Analyzer (Pocket PEA fluorimeter, Hansatech Instruments Ltd., England) as was previously described [75,80,123,124] after five days of seedling growth in the mini-hydroponic system described above at the following concentrations of methyl jasmonate: 15, 150, 500, 750 and 1000 µM (Figure S1). Leaves from ten plants from each of the three plastic containers were measured. One container was assumed to be one biological repetition. The abbreviations of the parameters that are used in the text are as follows: RC, the specific energy fluxes per reaction centre; CS, the specific energy fluxes per cross-section; ABS, the flux of the photons that were absorbed by the antenna pigments and that created excited chlorophyll; F_v_/F_m_, the maximum quantum efficiency of Photosystem II; PI_abs_, the Performance Index for the photochemical activity; ET_0_/CS, electron transport flux per CS; TR_0_/CS, trapped energy flux per CS; ABC/CS, absorption energy flux per CS; DI_0_/CS, dissipation energy flux per CS; and RC/CS, number of active reaction centres per illuminated cross-section [74,75,125].

The Nitrogen Balanced Index (NBI) was measured with a Dualex Scientifc (Force-A, France) sensor after five days of seedlings growth in medium with 500 µM methyl jasmonate (MeJA) and without the phytohormone supplement, i.e., the control growth conditions (Figure S1). The NBI index is the ratio of chlorophyll content to flavonoid content. Leaves from ten plants from each of the three plastic containers were measured. One container was assumed to be one biological repetition.

#### 4.3.2. Nitrogen Content Analysis

The nitrogen content in the plant tissue was analysed using the Total Kjeldahl Nitrogen method (TKN) [126] after five days of seedlings growth in a medium with 500 µM of methyl jasmonate (MeJA) and without the phytohormone supplement (i.e., the control growth conditions) in three biological repetitions (Figure S1). The leaves of the seedlings were cut and dried at 90 °C for three days. For each biological repetition, 0.5 g of the tissue was ground with an electric mill. To achieve this amount of tissue, the dried leaves of plants from 2–4 containers were polled, for the control growth or MeJA treatment conditions, respectively. After grinding, the plant material was dried for 24 h at 105 °C before the analysis. Then, 100 mg of the dried and homogenised samples were mineralised in H_2_SO_4_ with a catalyst at a temperature of 420 °C using a DKL42 Fully Automatic Digestion Unit (VELP Scientifica, Italy). The mineralised samples were distilled using a UDK129 Distillation Unit (VELP Scientifica, Italy) after adding 38% NaOH. During the distillation, the ammonium that had been obtained from most of the transformed nitrogen species evaporated as ammonia and then condensed in a 4% H_3_BO_3_ solution. Lastly, the distillates were titrated with 0.02 N HCl using a Tachiro indicator. A certified reference material (Hay Powder BCR^®^ 129, Institute for Reference Materials and Measurements, Belgium) was used for the quality assurance of the analytical data. The N concentration is presented as the g N kg^−1^d.w. The results are shown as the means ± SEs. The statistical significance of the differences was determined using a one-way ANOVA followed by the Fisher Least Significant Difference (LSD) test. Differences in the *p*-values < 0.05 were considered to be statistically significant. Statistica v. 13.1 (Dell Inc., USA) was used for the statistical analyses.

### 4.4. In Silico Analysis of HvTIP and the Oxygen-Evolving Complex (OEC) Genes in the Barley Seedlings

The members of the *TIP* gene subfamily in barley are already known [53]. The members of the *HvTIP* gene subfamily that are expressed in barley leaves at seedling stage growth were identified in our previous study [59]. The potential barley orthologues of Arabidopsis genes encoding the extrinsic proteins of the Oxygen-Evolving Complex (OEC) in the barley genome was identified using EggNOG 4.5.1—the database of the orthologous groups and functional annotations [127] (http://eggnogdb.embl.de/#/app/home). For a search in database, we used the Arabidopsis gene IDs and we identified barley MLOC numbers. Next, the MLOC number identified was used to search the Monocots Plaza database 4.5 (https://bioinformatics.psb.ugent.be/plaza/versions/plaza_v4_5_monocots/). Then, the identified sequences were used to perform a BLAST analysis against the barley genome sequence (reference) deposited in Ensemble Plants (https://plants.ensembl.org/Hordeum_vulgare/Info/Index) in order to identify the HORVU number for each gene. The Ensembl Plants *Hordeum vulgare* (IBSC_v2) was used.

### 4.5. qPCR Analysis of the HvMYC2 Gene, the Genes Encoding the Oxygen-Evolving Complex (OEC) and the Tonoplast Intrinsic Protein (HvTIP) Genes

The relative expression of *HvMYC2*, the *HvTIP* genes and genes encoding the extrinsic proteins of the Oxygen-Evolving Complex (OEC) were assessed using quantitative real-time RT-PCR (RT-qPCR) (Table S2) The total RNA was extracted from each barley sample using a TriPure Isolation Reagent according to the manufacturer’s protocol (Roche Life Science), which is based on the method of Chomczynski [128]. Before reverse transcription, 1 μg of the total RNA was treated with RNase-free DNase I (Fermentas) for 30 min in order to degrade any residual genomic DNA. Next, a RevertAid First Strand cDNA Synthesis Kit (Thermo Scientific) was used to synthesise the first-strand cDNA. The cDNA that was obtained was then diluted 1:5 with ddH_2_O and was used as the template for the quantitative PCR. The 10 µL of qPCR reaction mix contained 2.5 µL of diluted cDNA, 1 µL of the primer pair mixture (5 µM) and 5 µL of 2 × Master Mix (LightCycler 480 SYBR Green I Master; Roche). The RT-qPCR reactions were performed at 95 °C for 5 min followed by 45 cycles of 95 °C for 10 s, 58 °C for 20 s and 72 °C for 10 s. The value of the relative expression level was normalised to a reference gene *ADP* (*ADP-ribozylation factor 1*, accession no. AJ508228.2). The transcript level of the *HvMYC2*, genes encoding the extrinsic proteins of the Oxygen-Evolving Complex and the *HvTIP* genes in the leaves after 4, 24, 72 and 120 h of growth in the medium with 500 µM of MeJA, and the medium without the phytohormone supplement was calculated using the formula: Ct target gene – Ct reference gene (Figure S1). To analyse the expression after MeJA treatment, the relative expression of each gene at a given time point was determined as the fold change of its expression under the treatment conditions relative to its expression under the control conditions according to the delta-delta Ct method [129]. Three biological replications were used to analyse the gene expression using a sample of one seedling per replication. Each sample was analysed in two technical replicates. The relative expression data were analysed using the LinReq software tool [130], Statistica (13.1; Dell) and a one-way ANOVA followed by the Fisher Least Significant Difference (LSD); differences in the *p*-values < 0.05 were considered to be statistically significant.

## 5. Conclusions

Exogenous treatment with a high dose of methyl jasmonate (MeJA, 500 µM for 120 h) reduced photosynthesis efficiency in barley seedlings. The decrease of PSII parameters was associated with downregulation of *HvPsbR* gene encoding one of the extrinsic proteins of the Oxygen Evolving Complex. This, in turn, might lead to the impairment of OEC action, which was manifested by appearance of a K-band during analysis of fluorescence kinetics.

Methyl jasmonate treatment caused reduction of nitrogen content in barley leaves, associated with increased expression of four tonoplast aquaporin genes (*HvTIP1;2*, *HvTIP2;2, HvTIP4;1* and *HvTIP4;2*) predicted to transport nitrogen compounds from vacuole to cytosol. Upregulation of nitrogen-transporting *HvTIPs* may lead to vacuolar unloading of ammonia and urea, which both could be remobilised when nitrogen content decreased in barley leaves.

Drought and MeJA treatment led to the changes in expression profile of specific *HvTIPs* involved in water transport, indicating that activation of some aquaporin genes transporting water may be associated with MeJA pathway.

Changes in expression of all investigated *HvTIPs* in response to MeJA treatment are related to the presence of *cis*-regulatory elements in their promotor regions which are recognised by the jasmonate-related transcription factors.

## Figures and Tables

**Table 1:** Selected parameters related to photosynthesis efficiency measured in leaves of barley seedlings after MeJA treatment. Means ± for the SE are presented for each of the analysed parameters. Statistical analyses were performed using a two-way ANOVA (*p* < 0.05) followed by the Fisher Least Significant Difference (LSD) test (*p* < 0.05) to assess the differences between different concentrations of MeJA. Statistically significant differences (*p* < 0.05) are indicated by different letters (a–d). A decrease of a parameter value in relation to the control is indicated in blue and an increase in yellow. ABS/CS, absorption energy flux per cross-section (CS) of leaf area; TR_0_/CS, trapped energy flux per CS; ET_0_/CS, electron transport flux per CS; DI_0_/CS, dissipation energy flux per CS; RC/CS, number of active reaction centres per illuminated CS; PI_ABS_, performance index on absorption basis (based on [74]).

MeJA (µM)/ Parameter	ABS/CS	% of Control	TR_0_/CS	% of Control	ET_0_/CS	% of Control	DI_0_/CS	% of Control	RC/CS	% of Control	PI_ABS_	% of Control
control	1317.95 ± 9.38a	100	1032.96 ± 7.514a	100	578.89 ± 6.29ab	100	284.98 ± 2.22a	100	696.73 ± 6.83a	100	2.57 ± 0.052a	100
15	1341.43 ± 11.58a	101.8	1054.76 ±1 0.09a	102.1	615.81 ± 6.85b	106.4	286.67 ± 2.17a	100.6	764.54 ±1 0.70b	109.7	3.02 ± 0.075d	117.5
150	1271.83 ± 15.11ab	96.5	991.28 ± 12.05ab	96.0	552.62 ± 9.08a	95.5	280.54 ± 3.29a	98.4	665.40 ± 12.29a	95.5	2.44 ± 0.064a	94.9
500	1182.33 ± 13.89b	89.7	929.00 ± 11.81b	89.9	484.25 ± 9.61e	83.7	253.33 ± 3.12d	88.9	632.91 ± 13.47a	90.8	2.28 ± 0.074a	88.7
750	1008.83 ± 59.38d	76.5	782.01 ± 50.14d	75.7	387.58 ± 29.43d	67.0	226.82 ± 9.55c	79.6	498.45 ± 37.33c	71.5	1.74 ± 0.145c	67.7
1000	625.83 ± 58.18c	47.5	458.09 ± 48.44c	44.3	205.96 ± 25.63c	35.6	167.74 ± 10.30b	58.9	248.57 ± 29.66c	35.7	0.91 ± 0.116b	35.4

## Supplementary Materials

Supplementary materials can be found at https://www.mdpi.com/1422-0067/21/12/4335/s1: Figure S1. The mini-hydroponic system used to study the effect of methyl jasmonate (MeJA) treatment on seedling growth: Table S1. Potential orthologues of the Arabidopsis genes encoding the extrinsic proteins of the Oxygen-Evolving Complex in the barley genome; Table S2. Primers that were used in the qRT-PCR.

## Abbreviations

ABA	abscisic acid
ABS/CS	absorption energy flux per CS
APQ	aquaporins
ChIF	chlorophyll *a* fluorescence
CS	cross-section
DI_0_/CS	dissipation energy flux per CS
ET_0_/CS	electron transport flux per CS
MeJA	methyl jasmonate
MIP	membrane intrinsic proteins
NBI	nitrogen balanced index
OEC	oxygen evolving complex
PI_ABS_	performance index per absorption
PSI	photosystem I
PSII	photosystem II
PsbO/P/R/Q	photosystem II subunit O, P, R, Q
RC	reaction center
RC/CS	number of active reaction centers per illuminated cross-section
ROS	reactive oxygen species
TIP	tonoplast intrinsic proteins
TR_0_/CS	trapped energy flux per CS
