# Supplementary figures and images for: Methyl Jasmonate Affects Photosynthesis Efficiency, Expression of HvTIP Genes and Nitrogen Homeostasis in Barley
Source: Int J Mol Sci. 2020 Jun 18;21(12):4335. doi: 10.3390/ijms21124335 (PMC7352393; doi:10.3390/ijms21124335)

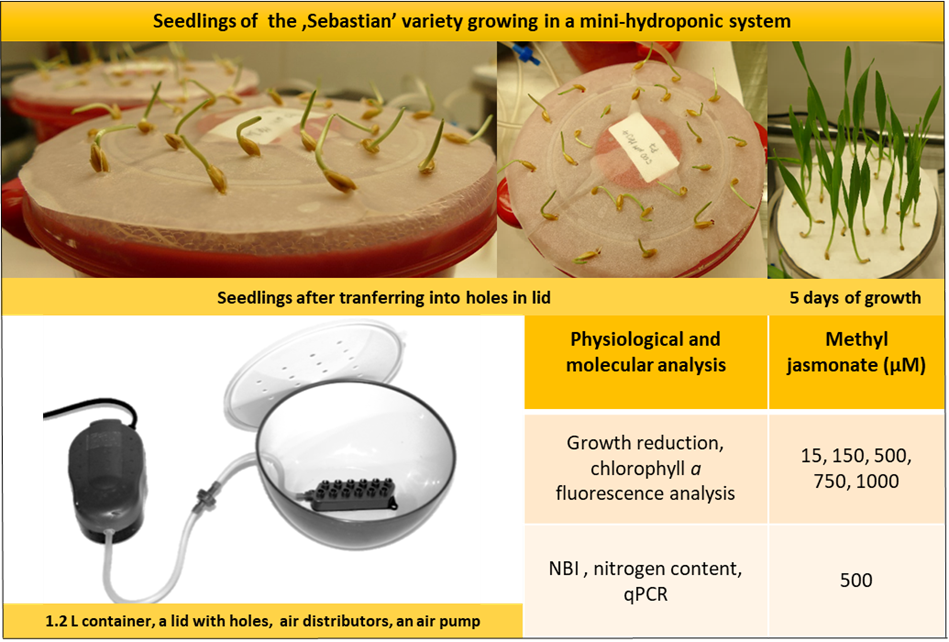

Supplement: Supplementary file 1 [file ijms-21-04335-s001.zip › Suppl. Figure 1.tif]

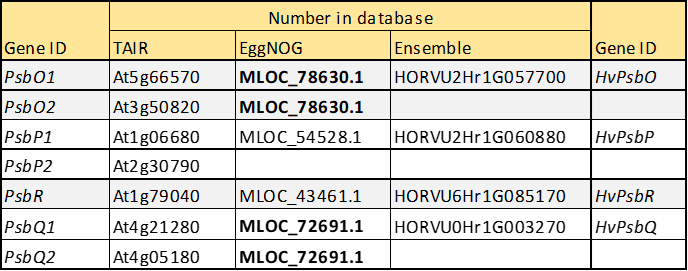

Supplement: Supplementary file 1 [file ijms-21-04335-s001.zip › Suppl. Table 1.tif]

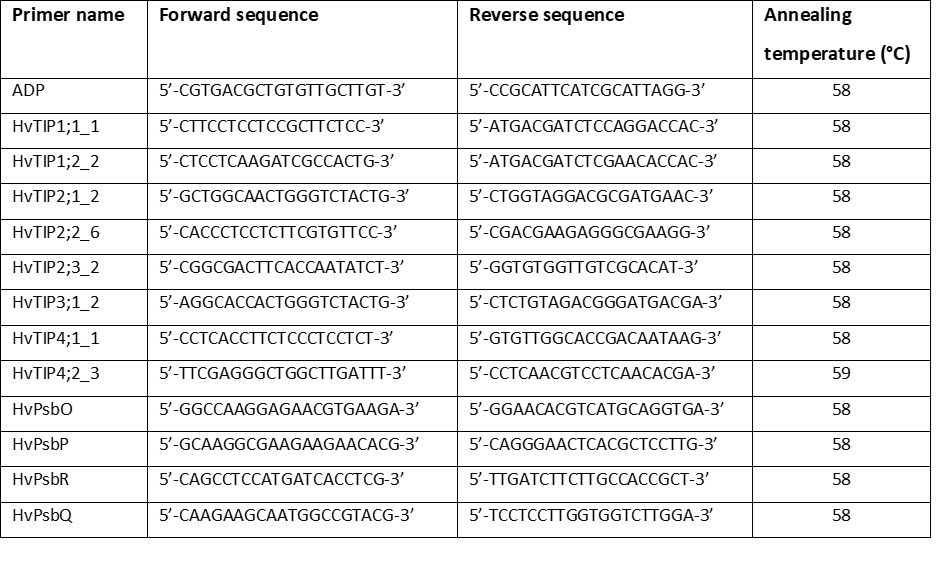

Supplement: Supplementary file 1 [file ijms-21-04335-s001.zip › Suppl. Table 2.tif]
